# Low altitude simulation without hypoxia improves left ventricular function after myocardial infarction by reducing ventricular afterload

**DOI:** 10.1371/journal.pone.0215814

**Published:** 2019-05-31

**Authors:** Anmol Shahid, Vaibhav B. Patel, Jude S. Morton, Trevor H. Stenson, Sandra T. Davidge, Gavin Y. Oudit, Michael S. McMurtry

**Affiliations:** 1 Dept. of Medicine, University of Alberta, Edmonton, Alberta, Canada; 2 Libin Cardiovascular Institute of Alberta, University of Calgary, Calgary, Alberta, Canada; 3 Dept. of Obstetrics and Gynecology, University of Alberta, Edmonton, Alberta, Canada; Scuola Superiore Sant'Anna, ITALY

## Abstract

Humans have a lower risk of death from myocardial infarction (MI) living at low elevations (<2500 m), which are not high enough to induce hypoxia. Both chronic hypoxia pre-MI, achieved by altitude simulation >5000 m, and intermittent hypobaric hypoxia post-MI can reduce MI size in rodents, and it is believed that hypoxia is the key stimulus. To explore mechanisms beyond hypoxia we studied whether altitude simulation <2500 m would also be associated with reduced infarct size. We performed left-anterior descending artery ligation on C57BL6 mice. Control mice (n = 12) recovered at 754 mmHg (atmospheric pressure, control), and treatment group mice (n = 13) were placed in a hypobaric chamber to recover 3-hours daily at 714 mmHg for 1 week. Echocardiographic evaluation of left ventricular function was performed on Day 0, Day 1 and Day 8. Intermittent hypobaric treatment was associated with a 14.2±5.3% improvement in ejection fraction for treatment group mice (p<0.01 vs. Day 1), with no change observed in control mice. Cardiac output, stroke volume, and infarct size were also improved in treated mice, but no changes were observed in HIF-1 activation or neovascularization. Next, we studied the acute hemodynamic effects of low altitude stimulation in intact mice breathing 100% oxygen using left ventricular catheterization and recording of pressure-volume loops. Acute reductions in barometric pressure from 754 mmHg to 714 mmHg and 674 mmHg were associated with reduced systemic vascular resistance, increased stroke volume and cardiac output, and no change in blood pressure or heart rate. Ex-vivo vascular function was studied using murine mesenteric artery segments. Acute reductions in barometric pressure were associated with greater vascular distensibility. We conclude that intermittent hypobaric treatment using simulated altitudes <2500 m reduces infarct size and increases ventricular function post-MI, and that these changes are related to altered arterial function and not hypoxia-associated neovascularization.

## Introduction

Myocardial infarction (MI) is very common and is associated with significant morbidity and mortality [1]. Most risk for MI is related to exposure to modifiable risk factors, including abnormal lipids, smoking, hypertension, diabetes, obesity, psychosocial factors, low consumption of fruits and vegetables, consumption of alcohol, and low levels of physical activity [2]. However, other exposures may modify the risk.

Epidemiological studies have consistently shown a significant reduction in mortality from MI in individuals living at low altitudes above sea level (<2500 m). Mortimer *et al*. reported a link between higher altitude and lower mortality from coronary artery disease in 1977, after evaluating mortality rates of Caucasian men in five counties in New Mexico at altitudes higher than 1220 m [3]. While this initial report was criticized as having confounding variables [4], subsequent studies have found similar associations between higher altitude of residence and lower mortality from coronary artery disease and stroke [5–7]. A larger study of 1198 Greek subjects identified a protective effect of living at high altitude (greater than 950 m) after adjustment for differences in other risk factors [8]. The largest study of altitude and cardiovascular disease included 1.64 million German-Swiss residents living between 259 and 1960 m, and included mortality data, social and demographic information, and places of birth and residence. The investigators demonstrated a relative risk reduction of ~22% per 1000 m of altitude above sea level for MI and ~12% per 1000 m for stroke [9]. Altitude is also associated with lower all-cause mortality in the general US population [10]. There appears to also be a link between higher altitude of residence and protection from death from MI for low elevations under 2500 m, however, the mechanism is not known.

Hypobaric hypoxic conditions mimicking high altitudes (>4000 m) have been studied as a method to limit infarct size when administered before or after MI in small mammals. Pre-conditioning of Wistar rats with hypobaric hypoxia simulating an altitude of 5000 m reduced infarct size by approximately 15% in an ischemia/reperfusion injury model [11]. In a rabbit MI model, 4 weeks of daily exposure to acute hypobaric hypoxia to a simulated altitude of 4000 m lead to an approximate 10% reduction in infarct size [12]. Similarly, exposing Sprague-Dawley rats after MI to intermittent hypobaric hypoxia corresponding to an altitude of 5000 m above sea level showed enhanced coronary flow, reduced left ventricular dilation, and improved left ventricular function vs. normoxic rats [13]. These reports suggest that altitude simulation to high altitudes, where there is a hypoxic stimulus, is beneficial post-MI. Whether there are additional mechanisms beyond a hypoxic stimulus, and whether altitude simulation post-MI to low elevations under 2500 m might be beneficial, are unknown.

We sought to determine whether normoxic low altitude simulation, or simulation to altitudes that do not cause hypoxia, can also reduce infarct size when administered after MI and to identify possible mechanisms.

## Materials and methods

### Ethical approvals

All protocols were approved by the University of Alberta Health Sciences Animal Care and Use Committee.

### Studying the effect of low altitude simulation on a mouse model of acute MI

#### LAD-ligation surgeries

Sixteen-week-old C57BL6 mice (Charles River; Wilmington, MA) were given access to standard chow and water ad-libitum and were housed on a 12h-12h light-dark cycle. Mice (n = 25) were anaesthetized through intubation with 2.5% isofluorane on a heated surgical platform, where a 3 mm incision was used to perform left- thoracotomy between the second and third ribs. The left anterior descending artery was located, and a 7–0 polyethylene suture was tied around the artery, immediately below the left atria without damaging the artery. The suture was tightened to completely and permanently restrict blood flow through the left anterior descending artery. 5–0 polyethylene sutures were used to carefully close the chest, layer by layer as previously described [14].

The animals were split into two groups, a control group (shelf controls; n = 12) and an experimental group (n = 13). Immediately following the surgery, the control group animals regained consciousness and recovered at room barometric pressure with access to standard chow and water ad-libitum. Animals in the experimental group regained consciousness and were immediately placed in a hypobaric chamber with a barometric pressure of 714 mmHg for a period of 3 hours. After the normoxic low altitude simulation treatment, these animals continued recovery at room barometric pressure with access to standard chow and water ad-libitum on a 12h-12h light-dark cycle. The experimental group of animals was administered normoxic low altitude simulation treatment (at 714 mmHg) daily for the next 7 days. The control group remained at atmospheric pressure, 754 mmHg, for the entire 7 days. Mice from another control group (chamber controls) were handled in the same manner as treatment animals and placed inside the hypobaric chamber for 3 hours daily for one week, except these animals were exposed to room atmospheric pressure (754 mmHg) while inside the chamber. All animals were monitored twice daily to ensure the animals recovered as expected post-operatively. As per recommendation of the Veterinarian with the University of Alberta Animal Care and Use Committee, Buprenorphine was used as an analgesic. After 7 days, the animals were sacrificed using cervical dislocation and tissue was harvested.

#### Echocardiography

Before and 24-hours following the LAD ligation surgery, echocardiographic assessment using the VEVO 2100 ultrasound system (Visualsonics, Toronto, Canada) was performed on all animals to verify that a major MI had occurred. Animals were anesthetized with 1.5% isoflurane in oxygen through the course of imaging, done with a high-frequency 40 MHz linear array transducer. The animals were placed on a heating pad to maintain 37°C body temperature and were constantly monitored through ECG through limb electrodes. We aimed to achieve heart rates of 420 beats per minute or above for the images acquired. Images obtained with physiological parameters that were suboptimal were strictly excluded from the analysis. The left ventricular chamber of all animals was imaged in parasternal long-axis and short axis views. Left ventricular M-mode imaging was conducted through tracing with the transducer at a sweep speed of 700 Hz at the papillary muscle level.

On day 7 following LAD-ligation surgery, echocardiography was performed on all animals to observe any changes in cardiac function that had occurred over the experimental period. Echocardiographic images were analyzed for standard parameters using the LV tracing method of M-mode images at the level of papillary muscles, corroborated by the Simpson’s method.

#### Altitude simulation

For normoxic low altitude simulation treatment, animals were placed inside of a custom constructed cylindrical 20.1 x 26.67 cm chamber (S1 Fig), sealable to maintain a lowered barometric pressure. A control pressure was established on the pressure sensor in relation to the barometric pressure denoted by the meteorological service of Canada (Edmonton, Alberta station). Low altitude simulation corresponding to a pressure of 714 mmHg, approximately 40 mmHg below the barometric pressure of the laboratory room was maintained through a vacuum controller (Buchi V-850; New Castle, DE) for a period of 3 hours daily with the experimental animals enclosed. This pressure was chosen to reflect a condition of low altitude, without inducing the effects of hypoxia on the animals.

### Histology and infarct size

To measure infarct size, hearts were excised from both groups of mice on Day 8 and flash frozen in OCT using liquid nitrogen. The hearts were sliced at the papillary level using a cryostat and stained with a standard protocol of Masson’s Trichrome Stain which stains cardiomyocytes red [15]. Infarct size was calculated as a percentage of the necrotic area versus area at risk.

#### Western blot analysis

Western blotting was performed with a standard technique using 25 μg of protein per sample obtained from peri-infarct cardiac tissue. We normalized expression to actin to correct for loading differences. The films were developed and quantified using the densitometry technique in the public domain Image J program (National Institutes of Health, MA, USA). The ImageJ “Gel Analysis” function was used, with background correction using a “rolling ball” method with a radius of 4 times the width of the band. The output was a value for each band proportional to the Integrated Density Value (IDV) of that band.

#### Lectin perfusion

All imaging was performed with an EVOS FL LED fluorescence microscope (Invitrogen, Waltham, MA, USA). 5 mg of lectin fluorescein ricinus communis agglutinin I (Vector Laboratories Inc, Burlingame, CA, USA) was injected via a central venous cannula for 5 minutes prior to sacrifice, cardiac tissue isolation, and flash freezing. Tissues were sliced 20 μm thick and fixed with 4% paraformaldehyde and imaged. For semi-quantification, peri-infarct zone fields were evaluated, and regions of interest were semi-quantified in arbitrary fluorescence units using Image J (National Institutes of Health, MA, USA). For quantification, the background was excluded from measured green fluorescence units with background correction using a “rolling ball” method with a radius of 50.0 pixels. The program was used to retrieve the RGB (red, blue, green) profile of the selected area, providing the intensity of each color in the region. Lectin perfusion was imaged in the green channel, hence the green intensity signal in the ROI (region of interest) was recorded for comparison of lectin perfusion between the control samples and treatment tissue samples.

### Studying the effect of low altitude simulation on the hemodynamics of intact mice

#### Pressure-Volume analysis

Left ventricular catheterization procedures were completed on male C57BL6 mice (aged 4–6 months old) using a FTH-1212B-4518 1.2 F admittance catheter (Transonic/SciSence; Toronto, ON) inserted via the carotid artery into the left ventricle as previously described [16]. The surgery was performed under 2.0% isoflurane anesthesia. The mouse was operated on inside of a custom constructed cylindrical 20.1 x 26.67 cm acrylic chamber (S2 Fig), sealable to maintain barometric pressure after catheter insertion into the left ventricle. LabChart 3 (ADInstruments; Sydney, Australia) provided real-time data and derived pressure-volume loops from several measured and calculated hemodynamic variables. The barometric pressure inside the chamber was recorded for 5 minutes at four pressure conditions (754 mmHg, 714 mmHg, 674 mmHg, and a return to 754 mmHg as per *ex vivo* protocols) and pressure-time relationships were recorded in the aorta before advancing the catheter through the aortic valve into the LV where pressure-time relationships were recorded again. The animal was euthanized through cervical dislocation following the protocol.

### Studying the effect of low altitude simulation on vascular function of isolated murine arteries ex vivo

#### Pressure myograph system

Sixteen-week-old C57BL6 mice (Charles River; Wilmington, MA) were given access to standard chow and water *ad libitum* and were housed in a 12h-12h light-dark cycle. Mice were euthanized through sodium pentobarbital administered intraperitoneally, and their mesenteries were removed and immersed in freshly prepared cold physiological salt solution (PSS: 10 HEPES, 5.5 glucose, 1.56 CaCl_2_, 4.7 KCl, 142 NaCl, 1.17 MgSO_4_, 1.18 KH_2_PO_4_ (in mM), pH 7.5). Two-second order resistance arteries were dissected from surrounding connective and adipose tissues and mounted in a pressure myograph system (Living Systems Instrumentation; Burlington, VT). Vessels were tied onto a glass cannula with a thin suture on either side such that the vessel was immersed in a PSS bath maintained at a temperature of 37°C. Intravascular pressure and flow were measured and alterable using pressure and flow control systems. A peristaltic pump was used to maintain specific rates of flow across the lumen of vessels mounted in the pressure myograph system. To mimic physiological conditions as best as possible, the vessels were oriented as such that flow would be applied in the same direction as *in vivo* blood flow. The vessels were equilibrated for 40 minutes during which the bathing PSS solution was changed multiple times. Ultimately, the vessels were maintained at a perfusion pressure of 60 mmHg to approximate the *in vivo* mesenteric arterial pressure of mice [17].

#### Drugs

All vessels were exposed to phenylephrine (PE) and methacholine (MCh) before the start of the experimental protocol to ensure the vessel was intact and capable of responding to pressure and flow stimuli. In some protocols, one bath of the pressure myograph was infused with inhibitors of NO synthase (L-NAME) and prostacyclins (meclofenamate) to inhibit the action of major endogenous endothelial vasodilators. This was done to study the effect of normoxic low altitude simulation on mesenteric arteries isolated from the vasoactive influences of nitric oxide and prostacyclins. In Ca^2+^ free protocols, 1x10^-4^ M papaverine was added to both baths (25 μl of 1x10^-2^ M in a 2.5 ml bath containing EGTA) to achieve maximal dilation for the blood vessels at an equilibrated transmural pressure of 60 mmHg. The drugs were added into the bath at room barometric pressure (754 mmHg) as the chamber needed to be opened for the addition. It took approximately 2 minutes to add drugs and reseal the chamber before hypobaric conditions were established again.

#### Low altitude simulation

The entire myograph system was enclosed within a custom designed 26.9 x 14.6 x 5.5 cm acrylic barometric pressure-controlled chamber (S3 Fig), capable of being sealed and pressure-controlled when necessary. A control pressure was established on the pressure sensor in relation to the barometric pressure denoted by the meteorological service of Canada (Edmonton, Alberta station). Pressure conditions of 714 mmHg and 674 mmHg were assigned as 40 and 80 mmHg below barometric pressure, respectively. Conditions were maintained through a vacuum controller (Buchi V-850; New Castle, DE).

A pressure transducer and flow regulator coupled with a peristaltic pump mechanism (Living Systems Instrumentation; Burlington, VT) was set up so it could be manipulated from outside upon sealing the barometric pressure chamber around the myograph system. The addition of any drugs in the myograph baths required opening of the chamber and thus bringing the vessels back to room barometric pressure. These drug additions were completed very efficiently to reduce the amount of time the chamber pressure was compromised (under 2 minutes). For the purposes of the experiment, three barometric pressures were simulated to mimic low altitude: 754 mmHg (control barometric pressure), 714 mmHg, and 674 mmHg. Pressure conditions were randomized to increase the validity of the experiments.

#### Measurements

Vessel diameter was recorded with the help of a micrometer coupled with a STEMI 2000 microscope (Zeiss; North York, ON). Measurements were taken after every intervention when the myograph had been sealed inside of the barometric pressure-controlled chamber. A transparent lid allowed for micrometer measurements. Desiccants lined the inner walls of the chamber to ensure visibility of the vessels was not diminished due to increased humidity in the chamber.

### Statistical analysis

All data are presented as mean ± SEM. For the *in vivo* MI data, we used the unpaired t-test for comparison between non-treatment and treatment groups where p<0.05 was considered significant. For the *in vivo* hemodynamic data, hypothesis testing was performed using one-way ANOVA with a p<0.05 considered significant. For the *ex-vivo* pressure myograph data, the significance of the difference in mean values of our continuous variables between groups was determined by a two-way ANOVA with a Bonferroni post-hoc test for multiple comparisons. We used the statistical software IBM SPSS Statistics 21 (Armonk, NY).

## Results

### Low altitude simulation treatment showed significant improvement in left ventricular function in post-MI mice

**Fig 1:**
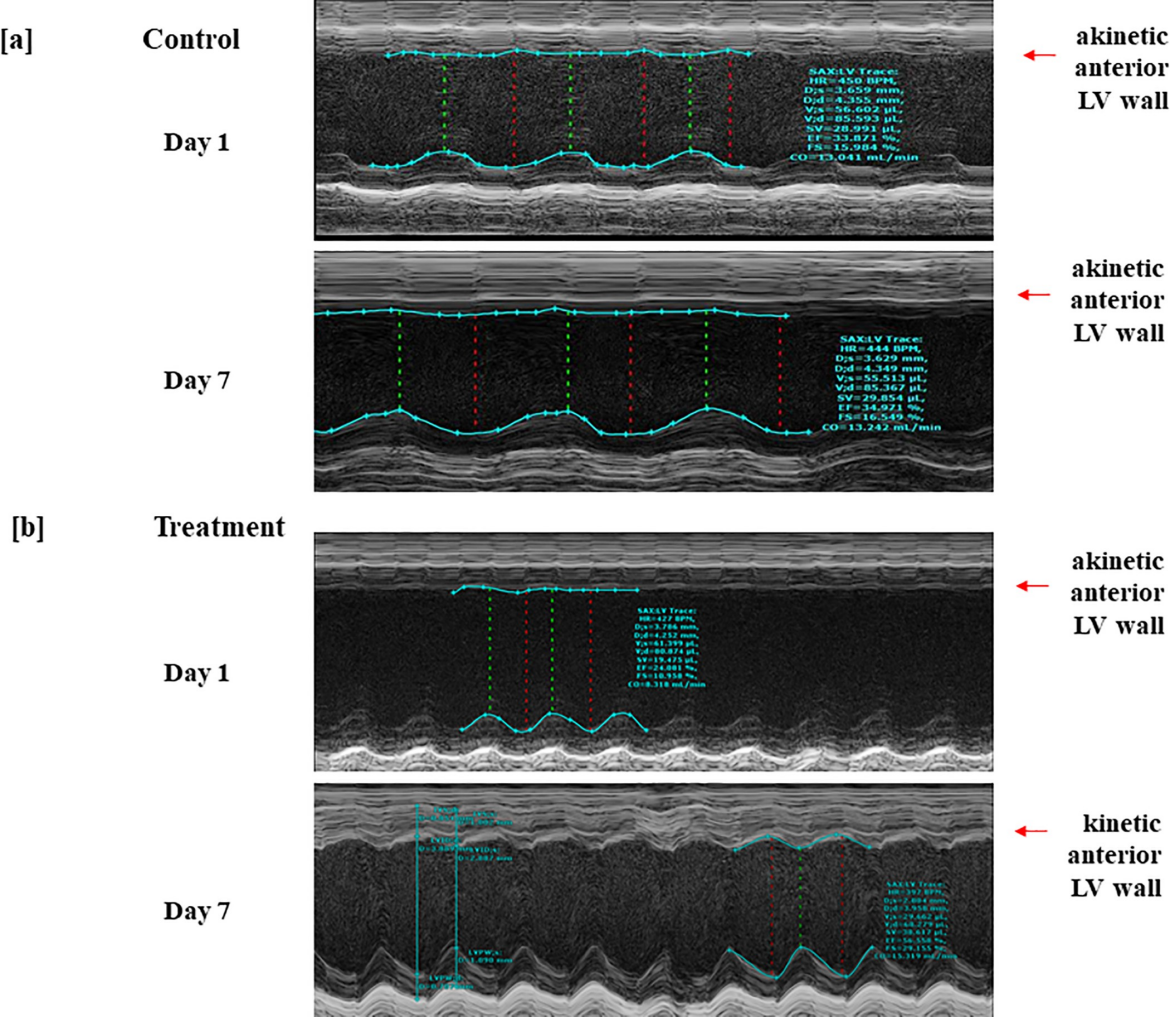
M-mode images were taken from animals that underwent LAD ligation and were treated with normoxic low altitude simulation in the custom constructed hypobaric chamber at 714 mmHg 3 hours daily for 7 days post LAD ligation surgery. The images shown are 24 hours after the LAD ligation in animals that recovered at room conditions (a) and those treated with 714 mmHg for 7 days (b). There is marked akinesis of the left anterior left ventricular wall at Day 1. At Day 7, this akinesis is replaced with dramatically improved movement of the anterior LV wall.

After LAD ligation surgery animals were assigned to 7 days of normoxic low altitude treatment in the custom-built hypobaric pressure chamber (S1 Fig) or no treatment. Echocardiographic M-mode data obtained on Day 1 of the study post LAD ligation showed a clear akinesis of the anterior wall of the left ventricle in both groups of animals (Fig 1 and S4 Fig) in comparison to baseline images taken the day before surgery. Upon repetition of the echocardiogram after 7 days, the shelf and chamber control animals that were left to recover at room barometric pressure of 754 mmHg showed no improvement in the function of their anterior left ventricular wall (Fig 1A). However, animals treated with the altitude simulation of 714 mmHg for a period of 7 days after the LAD ligation surgery showed a very noticeable movement in their anterior LV wall (Fig 1B). These M-mode images were carefully taken at the papillary muscle level of the left ventricle in short axis orientation to ensure imaging of the correct section of the ventricle.

**Fig 2:**
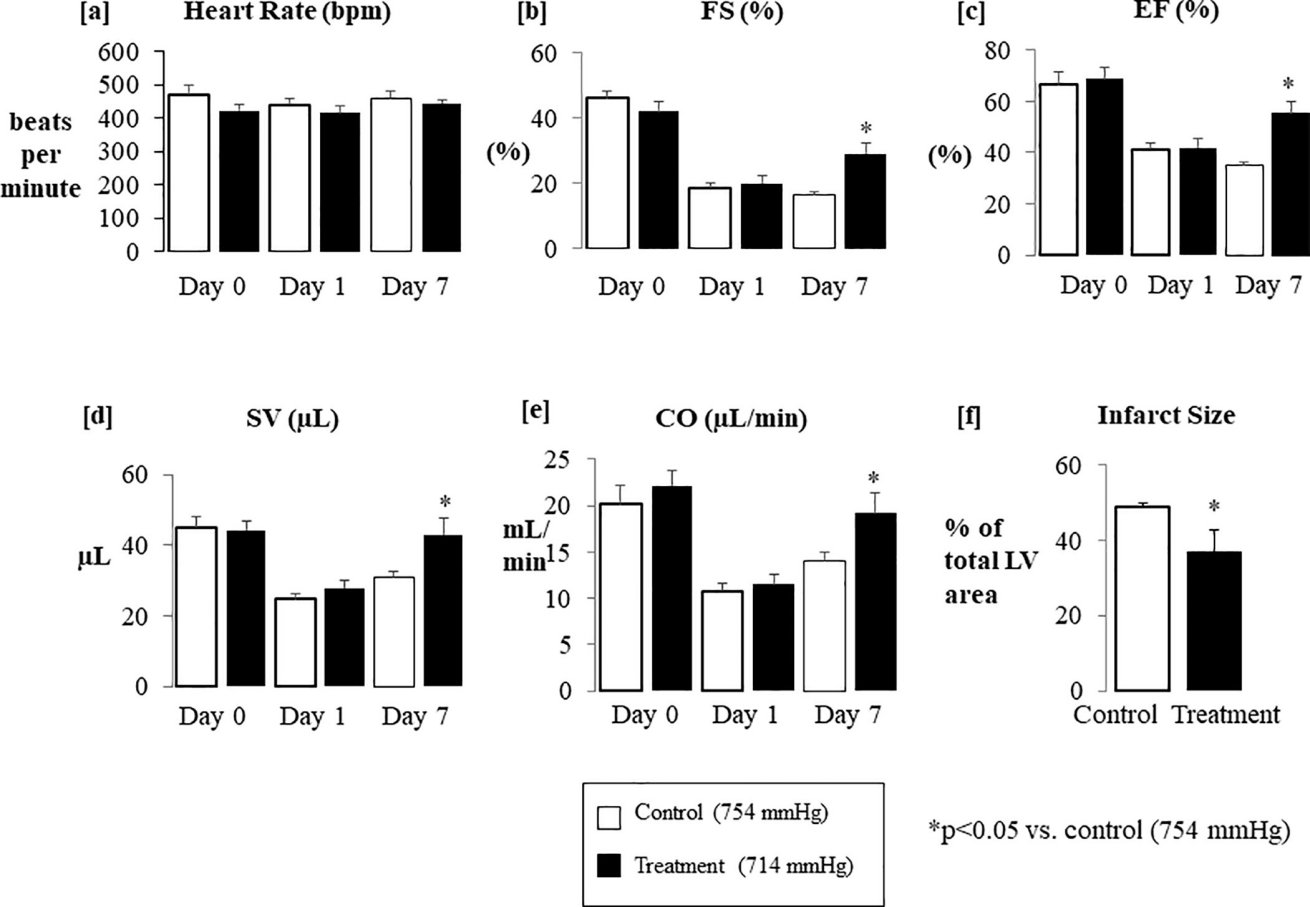
Hemodynamic parameters were obtained through echocardiography for experimental and untreated animals at Day 0 along with Day 1 and Day 7 after LAD ligation surgery. There were no significant differences in heart rate (a) in both sets of animals from Day 1 to Day 7. There were statistically significant increases in fractional shortening (b), ejection fraction (c) stroke volume (d) and cardiac output (e) in animals that received 3 hours of normoxic low altitude simulation (at 714 mmHg) for 7 days in comparison to shelf and chamber control animals. Improvements in left ventricular function as shown by these parameters were absent from animals that did not receive normoxic low altitude simulation treatment.

Through echocardiographic imaging of the animals in parasternal long axis mode and short axis mode at the apical, papillary, and base of the left ventricle, we quantified standard cardiac function parameters (Fig 1) using the Simpson’s method for accuracy and reproducibility. We found no significant differences in heart rate (Fig 2A) of the shelf and chamber control vs. treatment animals at Day 1 or Day 7 after the LAD ligation surgery. Both controls and treatment group animals started with similar fractional shortening measurements at Day 1. However, at Day 7, the animals that had been treated with normoxic low altitude simulation (at 714 mmHg) showed an improvement in their fractional shortening of 9.3±3.2%, representing an approximate 50% increase in function between days 1 and 7 (Fig 2B). Similarly, controls and treatment group animals started at similarly diminished levels of ejection fraction Day 1 after the LAD ligation. At Day 7, however, treatment animals showed a 14.1±5.1% increase in their ejection fraction values compared to animals who did not receive normoxic low altitude simulation treatment (Fig 2C). Stroke volume and cardiac output measurements in animals that received normoxic low altitude simulation treatment (at 714 mmHg) were significantly improved compared to the shelf and chamber control animals that recovered at room barometric pressure of 754 mmHg for a period of 7 days. Increases in stroke volume of 12.7±5.2 μL (Fig 2D) and an increase of 8.6±2.38 μL/min (Fig 2E) were observed. Day 1 and Day 7 treatment group data shown alongside data from both chamber and shelf control groups (S5 Fig) reveals no significant differences between the two control groups.

### Normoxic low altitude simulation treatment reduced infarct size in mice after LAD ligation surgery

At Day 7 after the LAD ligation surgery, the hearts were excised and stained with Masson’s Trichrome stain. Animals that received normoxic low altitude simulation treatment after the MI had significantly reduced infarct size compared to animals that did not receive treatment (Fig 2F). A reduction of 23.6±6.4% in infarct size was observed between the group treated with normoxic low altitude simulation (714 mmHg) vs. the group that received no treatment when normalized to a “no infarct” image.

### Normoxic low altitude simulation treatment reduced HIF-1α expression in the peri-infarct tissue of mice after LAD ligation surgery

**Fig 3:**
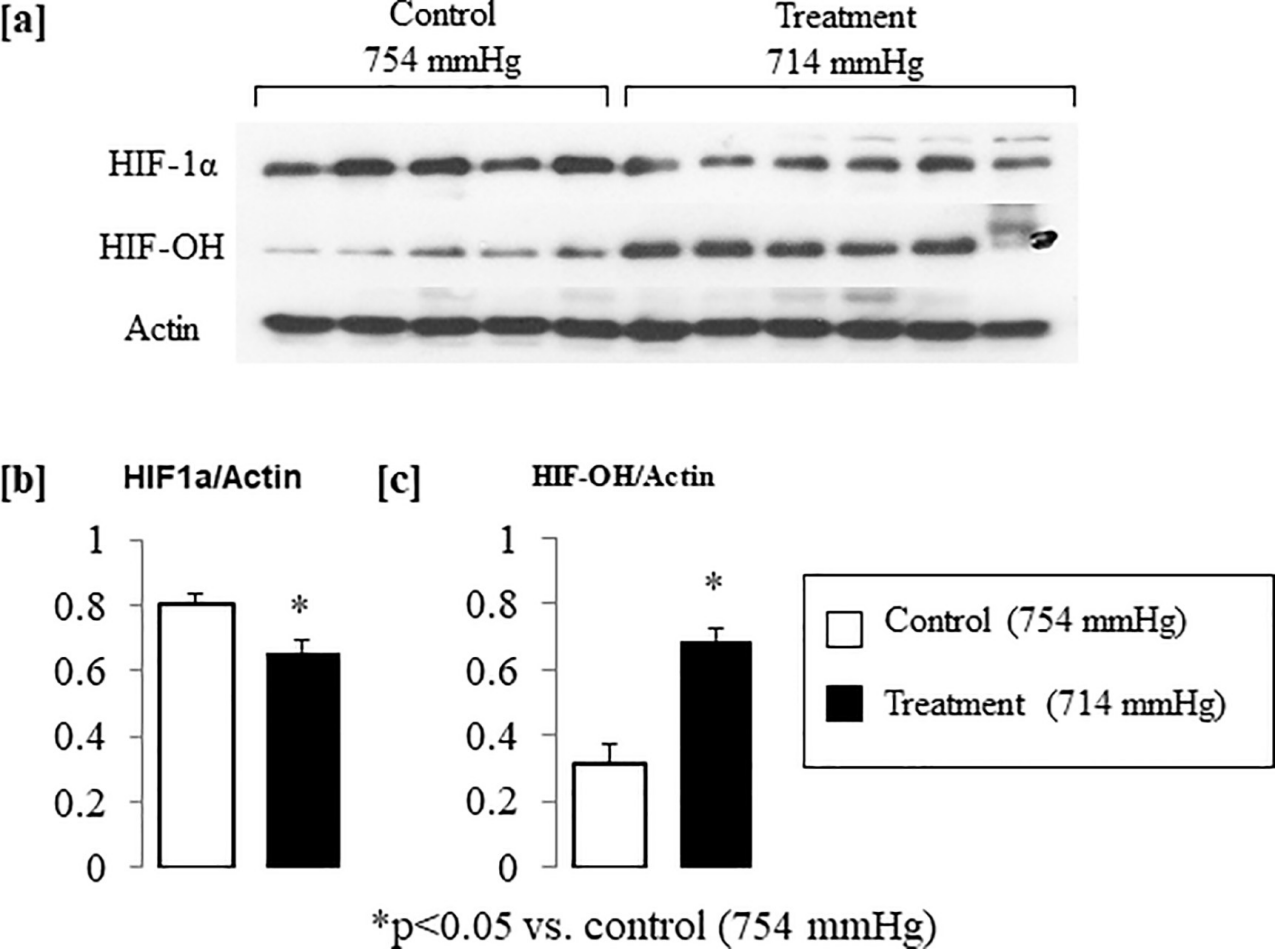
**Western blot staining showed decreased HIF-1α expression in peri-infarct tissue obtained from treatment animals vs. control animals (a,b).** HIF-1α-OH shows an opposite trend, with significantly more degraded HIF-1α in treatment animals vs. control animals (c). The values were normalized to actin to control for loading differences and analyzed with ImageJ Software.

Western blot analysis of peri-infarct tissue excised from select animals after Day 7 of the study showed a significantly reduced expression of HIF1α in animals that received normoxic low altitude simulation treatment (at 714 mmHg) for 7 days after LAD ligation surgery (Fig 3A and 3B). This trend was reversed for HIF-1α-OH expression, which was significantly higher in animals that were treated with normoxic low altitude simulation for 7 days after the LAD ligation surgery (Fig 3A and 3C). All bands were normalized to actin expression to correct for loading differences in the gel.

### Normoxic low altitude simulation treatment was not associated with significant differences in lectin perfusion of peri-infarct tissue areas between treatment animals and control animals

**Fig 4:**
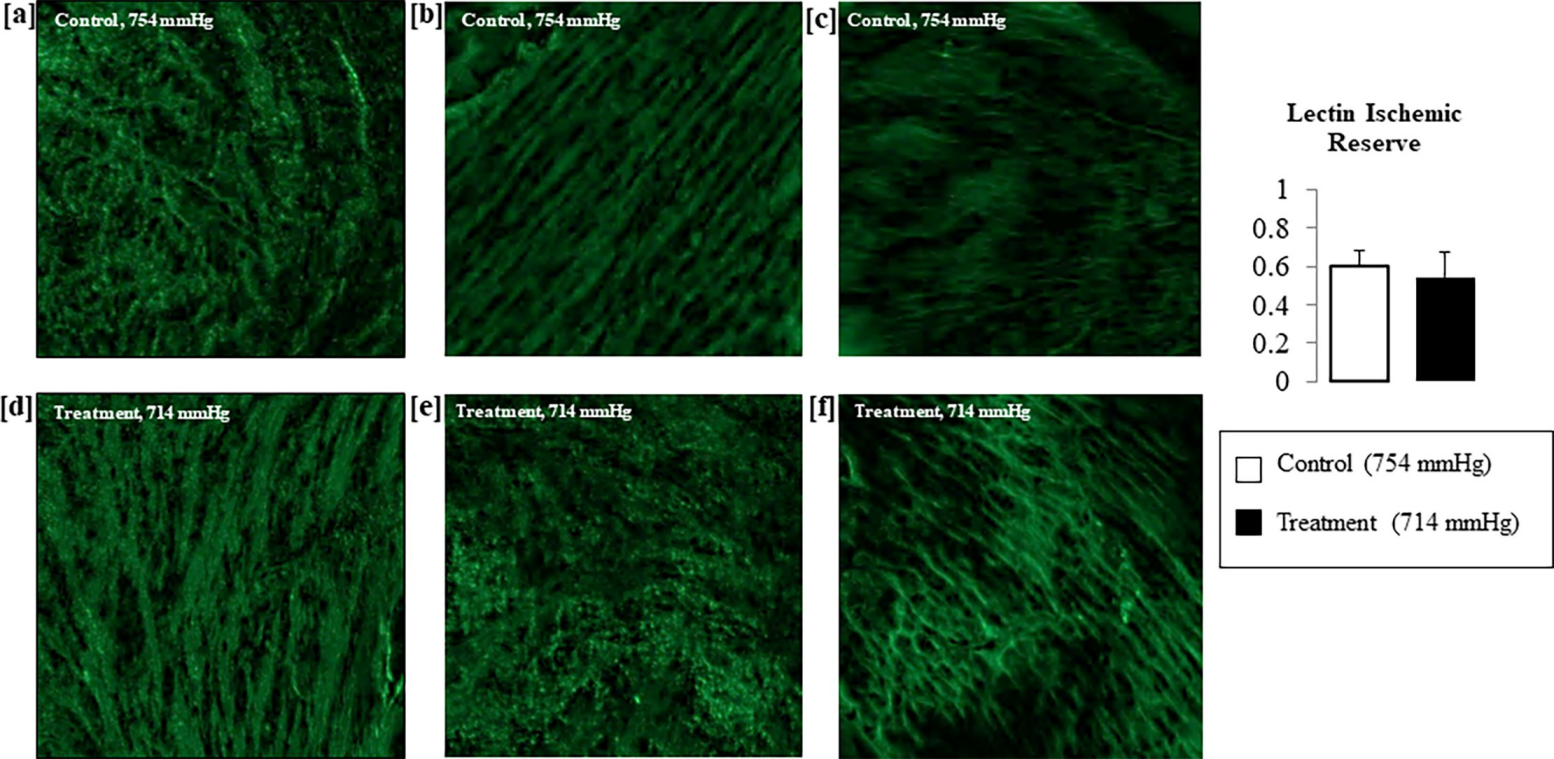
Lectin perfusion staining shows no significant difference in perfusion in peri-infarct tissue obtained from normoxic low altitude simulation treatment animals (a-c) vs. control animals (d-f).

After a period of 7 days post LAD ligation surgery, lectin was perfused into select animals before excision of hearts for lectin perfusion analysis. The analysis showed no significant differences in the lectin perfusion (as measured by green signal) in the heart tissue of animals that did not receive normoxic low altitude simulation (Fig 4A, 4B and 4C) vs. treatment animals that received 7 days of normoxic low altitude simulation treatment at 714 mmHg (Fig 4D, 4E and 4F). The lectin ischemic reserve, an index of lectin perfusion, was not different between the two groups of animals (Fig 4E).

### Normoxic low altitude simulation is associated with increased stroke volume, increased cardiac output, and decreased total systemic vascular resistance in freely breathing healthy mice in vivo

**Fig 5:**
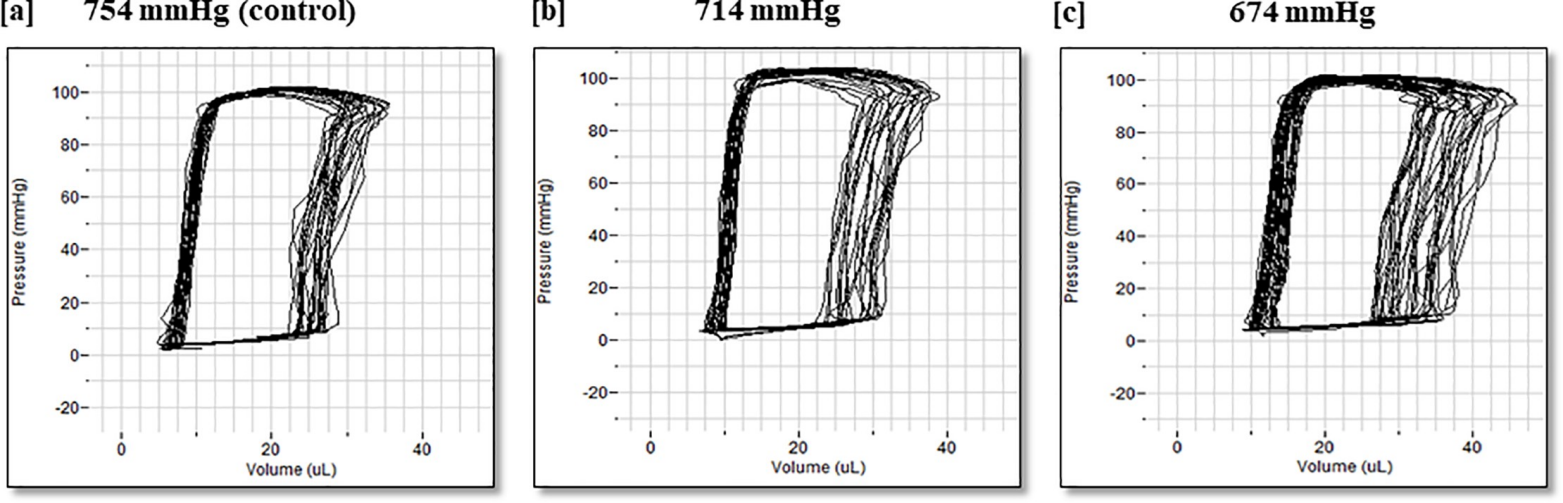
**Pressure-volume loops were obtained through invasive left-ventricular catheterization *in vivo* at normoxic low altitude simulation conditioning corresponding to barometric pressures of 754 mmHg (a), 714 mmHg (b), and 674 mmHg (c).** Values shown are means ± SE.

**Fig 6:**
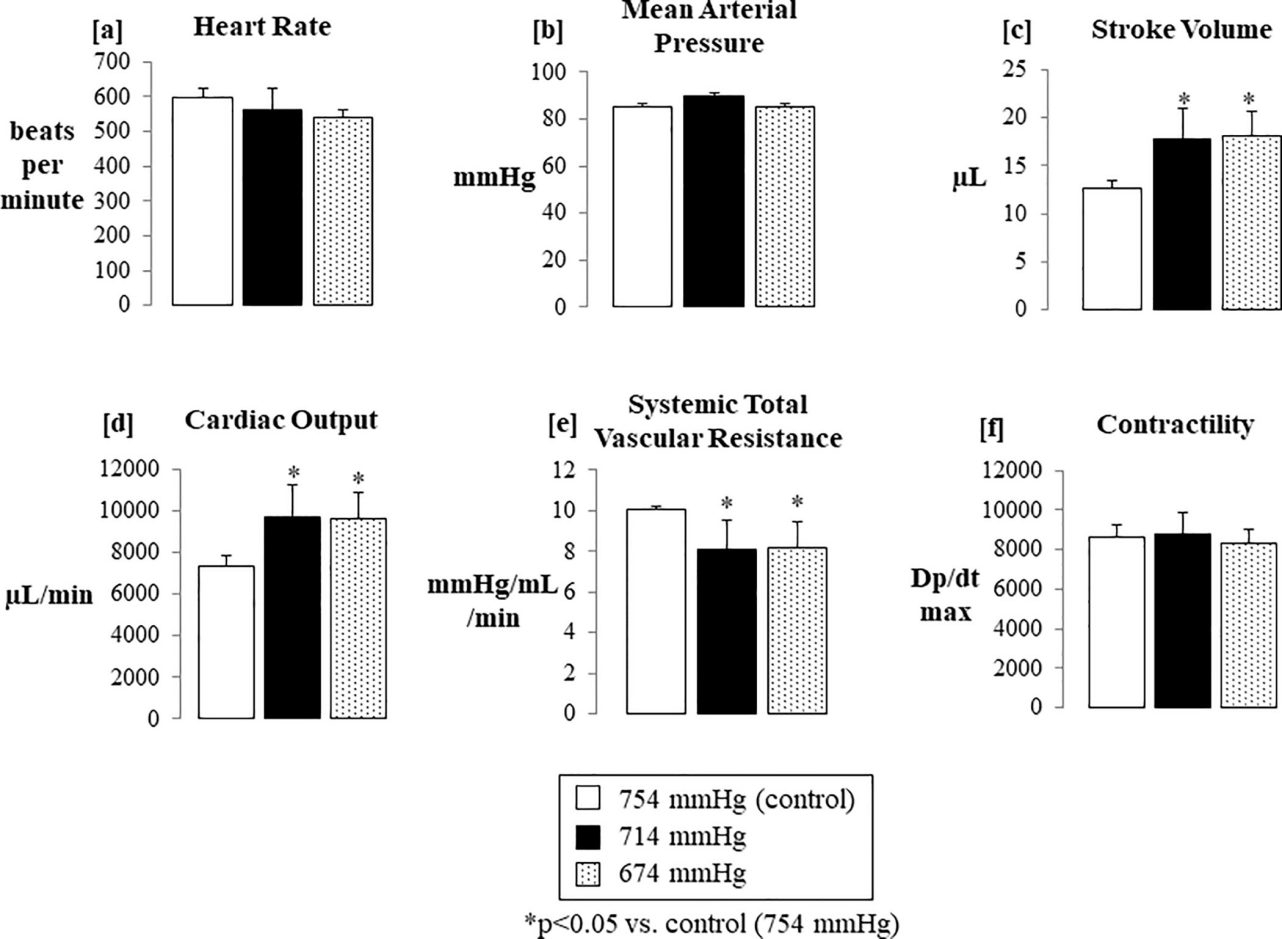
Hemodynamic parameters were obtained through invasive left-ventricular catheterization *in vivo* at normoxic low altitude simulation corresponding to barometric pressures of 754 mmHg, 714 mmHg, and 674 mmHg. There were no significant differences in heart rate (a) or mean arterial pressure (b). There were statistically significant increases in stroke volume (c) and cardiac output (d). Calculated total systemic vascular resistance was also reduced by normoxic low altitude simulation (e). Left ventricular contractility, as measured by dP/dt, was not changed by normoxic low altitude simulation (f). Values shown are means ± SE.

The pressure-volume relationships obtained via invasive catheterization of the left ventricle through the carotid artery in a closed chest procedure at 754 mmHg (Fig 5A), 714 mmHg (Fig 5B), and 674 mmHg (Fig 5B) show a rightward shift of the pressure-volume relationship seen after 5 minutes of exposure to normoxic low altitude simulation corresponding to 714 mmHg (Fig 5), with a further right-skewing and a decreased systolic blood pressure trend with exposure to normoxic low altitude simulation corresponding to 674 mmHg (Fig 5C). The parameters shown in Fig 6A–6F are measured and calculated values from the inserted catheter. Invasive pressure-volume hemodynamic analyses showed preserved mean arterial pressures and dp/dt max (Fig 6B and 6F) across the low altitude simulation corresponding to barometric pressure conditions of 754 mmHg, 714 mmHg, and 674 mmHg. Stroke volume (SV) (Fig 6C) and cardiac output (CO) (Fig 6D) were increased significantly (n = 9, p<0.05) at 674 mmHg and 714 mmHg as compared to 754 mmHg. SV and CO indices returned to near baseline values when normoxic low altitude simulation was ceased at the end of each protocol, demonstrating the transient nature of the induced effects.

### Normoxic low altitude simulation increased arterial distensibility under flow manipulation conditions in murine resistance arteries

**Fig 7:**
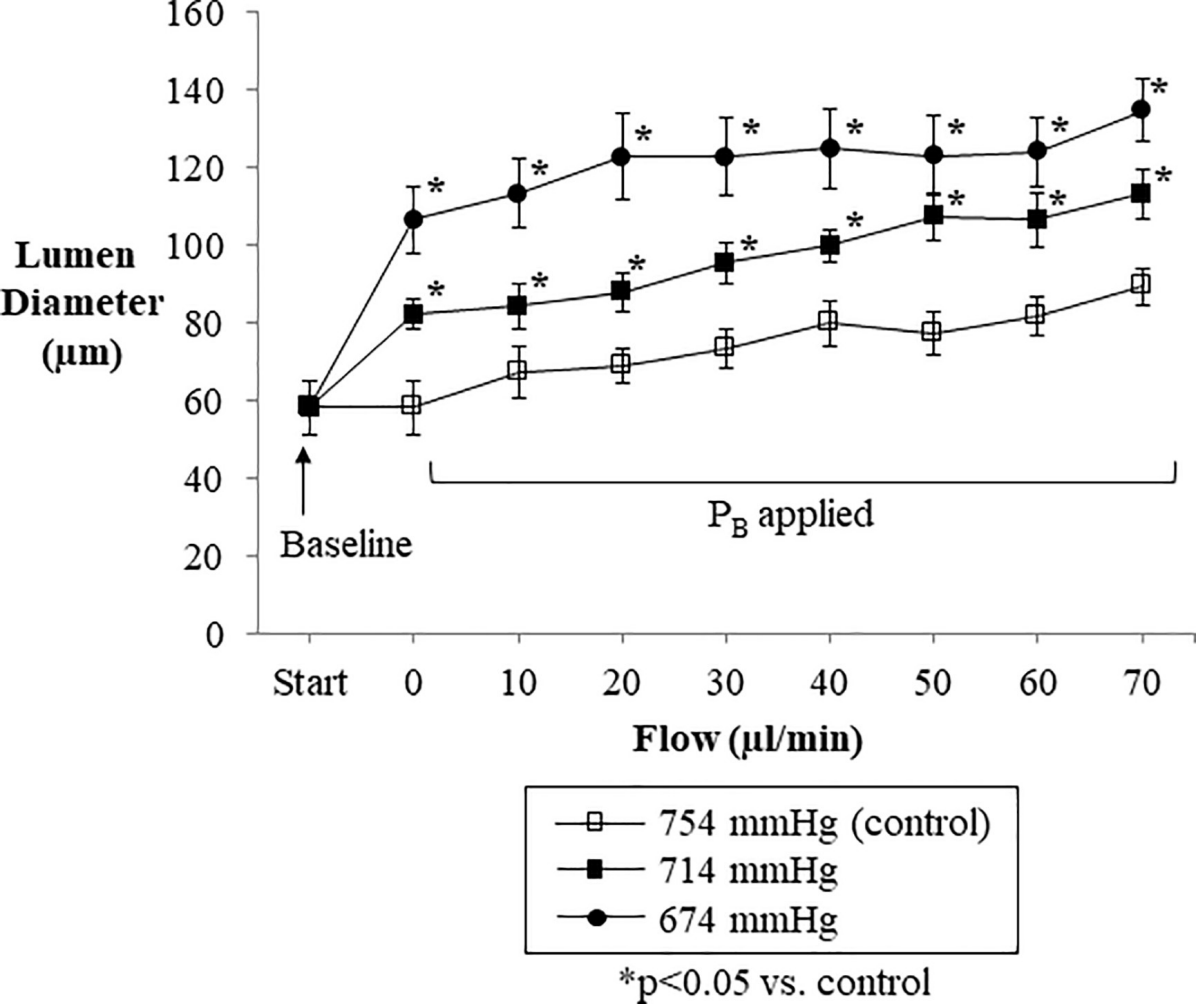
Segments of murine mesenteric artery were placed in a pressure myograph which was in turn placed within a specially constructed chamber to induce normoxic low altitude simulation corresponding to various barometric pressures. The lumen diameter of perfused segments of murine mesenteric artery increased as the internal flow through the arterial segment increased across a range of physiologically relevant flow volumes (b). Normoxic low altitude simulation corresponding to reductions barometric pressure around the perfused arterial segments from 754 mmHg to 714 mmHg and 674 mmHg significantly augmented the lumen diameter response to increased flow volume. Values shown are means ± SE.

Murine second order mesenteric arteries (n = 8) mounted on a pressure myograph (S3 Fig) responded with increased lumen diameter across a range of physiologically relevant flow rates (Fig 7), as expected. The same arterial segments responded with significantly greater lumen diameters under normoxic low altitude simulation to mimic either a barometric pressure of 714 mmHg or 674 mmHg, with the larger barometric pressure reductions associated with larger increases in lumen diameter across a range of flow rates. Lowering barometric pressure (i.e., simulating altitude) showed an immediate increase in arterial diameter even at very low flow rates. A very large increase in vessel diameter was seen with normoxic low altitude simulation corresponding to 674 mmHg across all flow rates.

### Normoxic low altitude simulation enhanced passive and active pressure mediated increase of arterial diameter in murine resistance arteries

**Fig 8:**
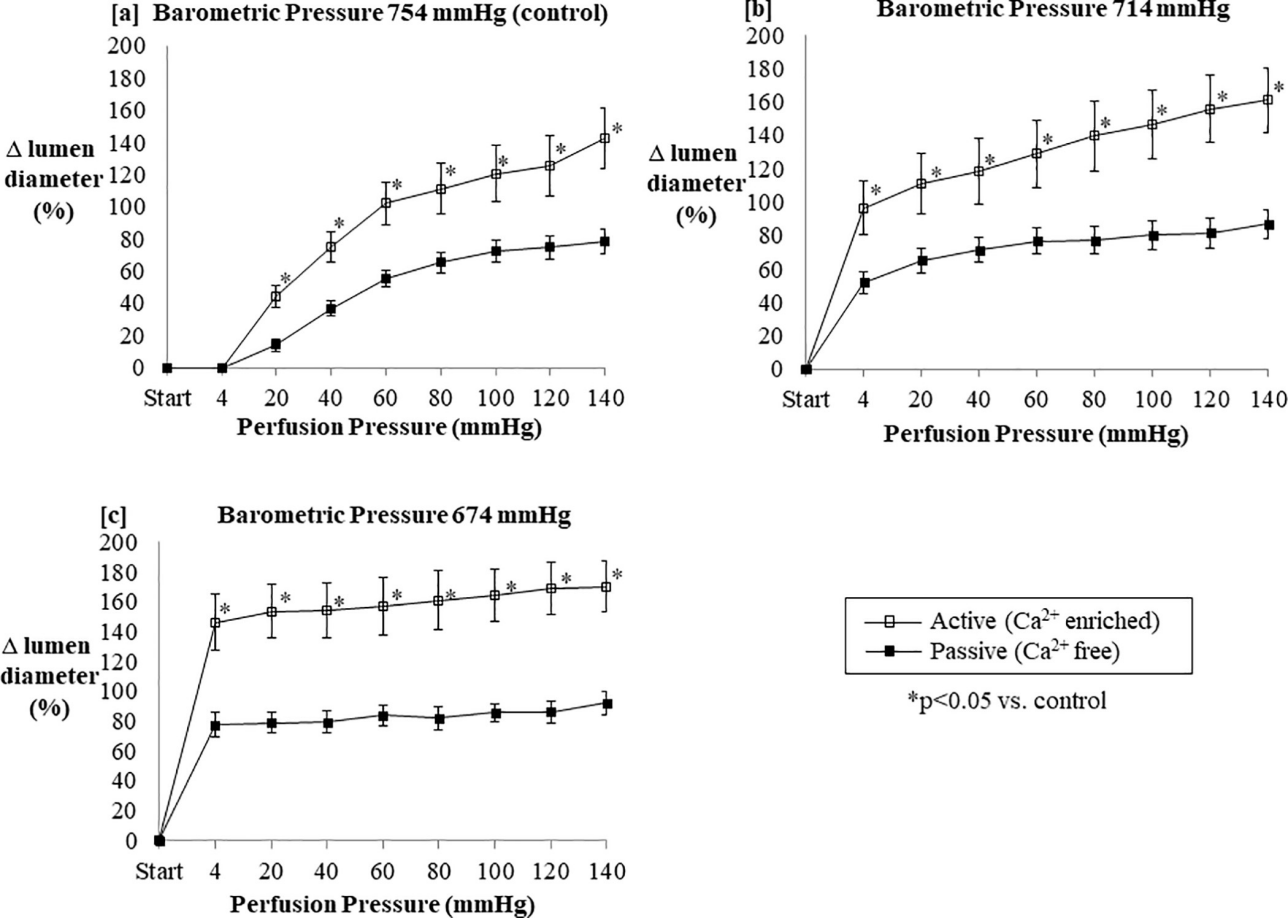
The lumen diameter of perfused segments of murine mesenteric arteries increased as internal perfusion pressure increased across a range of physiologically relevant perfusion pressures for both Ca^2+^-free and Ca^2+^-enriched conditions across increasing altitude simulation. Normoxic low altitude simulation around the perfused arterial segments corresponding to barometric pressures from (a) 754 mmHg to (b) 714 mmHg and (c) 674 mmHg significantly augment the lumen diameter response to increased internal perfusion pressure, especially in calcium enriched vessels. Values shown are means of percent changes in lumen diameter ± SE.

Murine second order mesenteric arteries mounted on a pressure myograph in calcium-enriched and Ca^2+^ free conditions (n = 7) responded with increased lumen diameter across a range of physiologically relevant intraluminal pressures (Fig 8A), with arteries in Ca^2+^ free solution showing a lesser increase in lumen diameter. However, at a very slight altitude simulation corresponding to a barometric pressure of 714 mmHg (Fig 8B), initial arterial diameter change was larger at the start of the protocol. This effect persisted in both active and passive conditions. An even greater increase in starting lumen diameter was observed upon normoxic low altitude simulation corresponding to a barometric pressure at 674 mmHg (Fig 8C). normoxic low altitude simulation was associated with increased lumen diameter, with the greatest changes in lumen diameter observed at the lowest perfusion pressures right after the application of normoxic low altitude simulation. Although normoxic low altitude simulation was associated with dose-dependent increases in lumen diameter, the magnitude of increases in lumen diameter were somewhat smaller at a given perfusion pressure for the arteries in the absence of physiological Ca^2+^ versus arterial with intact arterial tone (active).

### Increases in murine resistance arterial lumen diameter in response to normoxic low altitude simulation are not significantly attenuated by the presence of inhibitors of endothelial function

**Fig 9:**
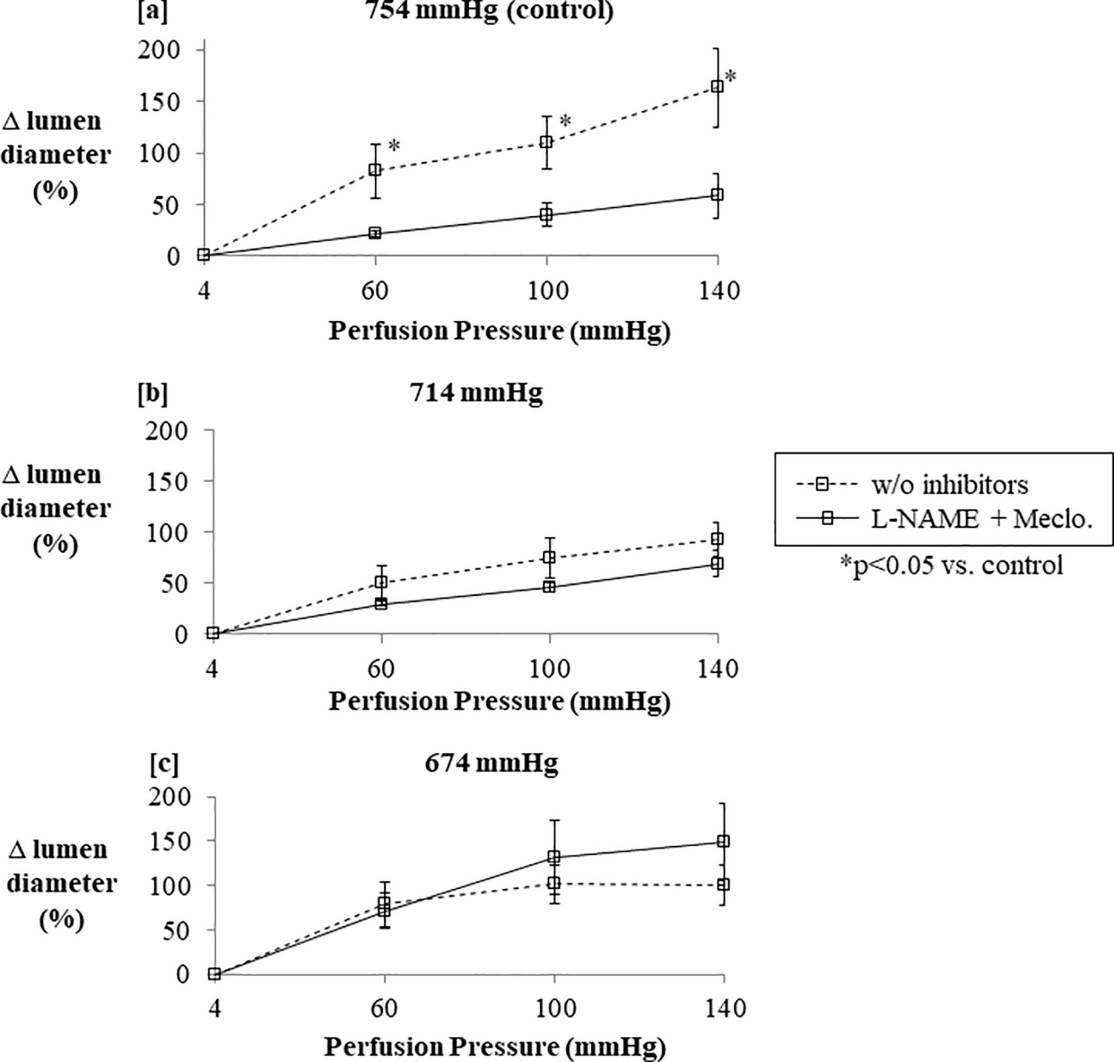
**In perfused segments of the murine mesenteric artery at room barometric pressure, the percent distension from baseline in response to increased perfusion pressure is higher in the absence of inhibitors of endothelial function (a), supporting that the increase in vessel diameter is in part endothelium-dependent.** However, when normoxic low altitude simulation surrounding the perfused arterial segment is applied to generate barometric pressures from 754 mmHg to 714 mmHg (b) and 674 mmHg (c), arterial segment percentage diameter increase is similar with or without inhibitors of endothelial function, supporting that endothelium contributes less to the diameter increase under these conditions. Values are the mean percentage change in lumen diameter ± SE.

Murine resistance arteries mounted on a pressure myograph and at room barometric pressure (n = 8) responded with increased lumen diameter across a range of perfusion pressures, but the presence of inhibitors of endothelial function, including L-NAME and meclofenamate, attenuated the maximal percentage increase in lumen diameter achieved from 163.5±21.1% to 58.6±27.1% (p < 0.05) (Fig 9A). Under conditions of normoxic low altitude simulation, however, the same murine resistance arteries demonstrated similar percentage increases in lumen diameter regardless of whether L-NAME and meclofenamate were present (Fig 9B and 9C).

### Murine resistance artery lumen diameter varied directly with dose-dependent increases in methacholine and normoxic low altitude simulation

**Fig 10:**
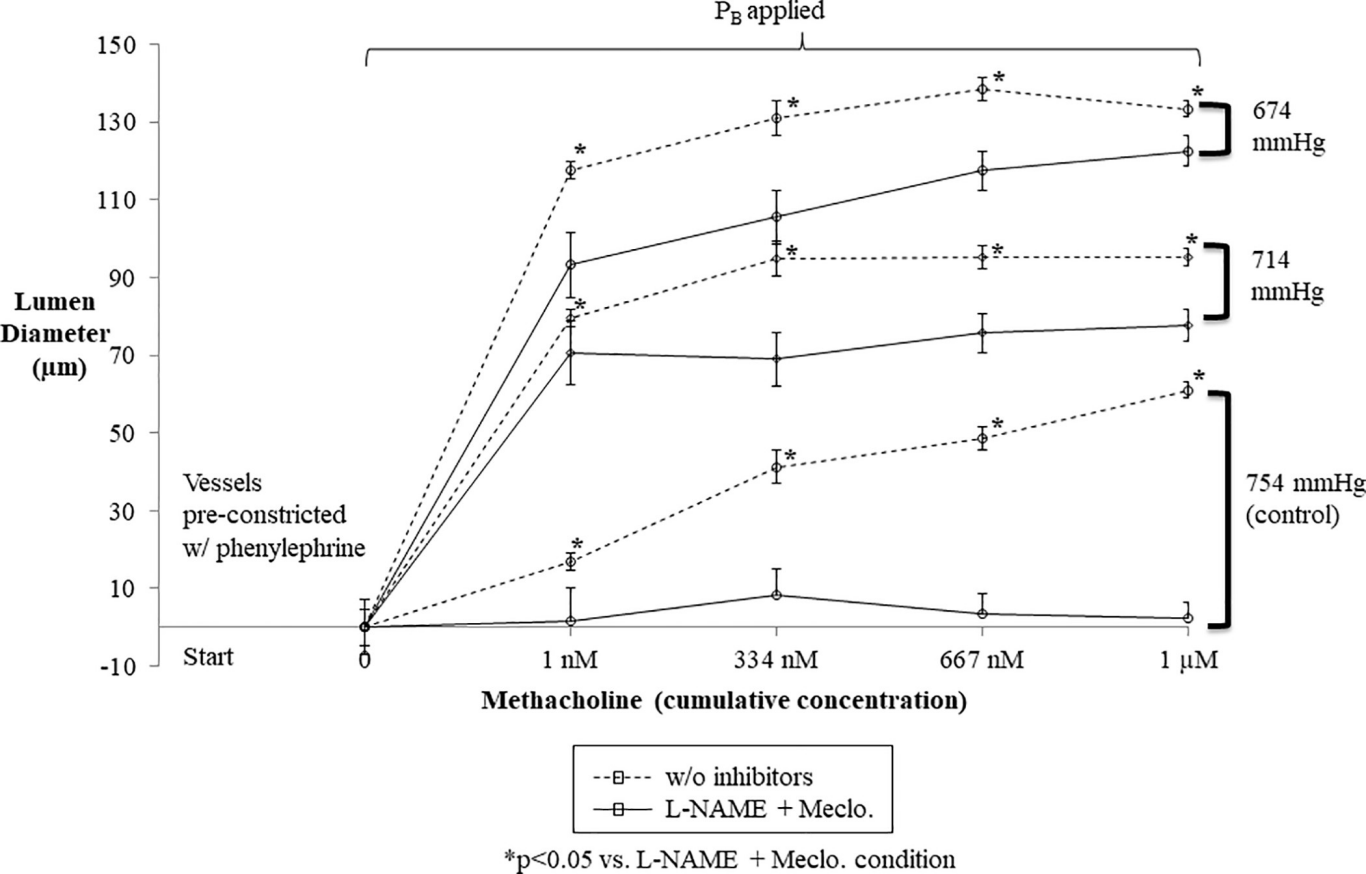
In pre-constricted perfused segments of the murine mesenteric artery, increasing doses of methacholine significantly increased lumen diameter in the absence of inhibitors of endothelial function, regardless of normoxic low altitude simulation (at 714 mmHg or 674 mmHg). However, normoxic low altitude simulation surrounding the perfused arterial segment to generate pressures of 714 mmHg and 674 mmHg substantially increased lumen diameter, even more than maximal doses of methacholine at 754 mmHg. Values shown are means ± SE.

At room barometric pressure (754 mmHg), murine resistance arteries mounted on a pressure myograph (n = 12) responded with increasing lumen diameters as increasing doses of methacholine were added to the bath (Fig 10). In the same murine resistance arteries, normoxic low altitude simulation augmented lumen diameter in a dose-dependent manner. The application normoxic low altitude simulation to generate barometric pressure to 714 mmHg and 674 mmHg induced increases in lumen diameter greater than any dose of methacholine alone at room barometric pressure (754 mmHg). Highly dilated lumen diameters were observed with the normoxic low altitude simulation corresponding with a barometric pressure of 674 mmHg and the highest doses of methacholine.

## Discussion

To explore why patients living at low altitudes that are not associated with hypoxia have less mortality from myocardial infarction, we conducted a set of experiments to determine whether normoxic low altitude simulation after the induction of a severe MI in mice could improve myocardial function and reduce infarct size. We found of improvements in fractional shortening, ejection fraction, stroke volume, and cardiac output with 7 days of normoxic low altitude simulation treatment in mice with severe MI, consistent with altitude exposures to higher elevations associated with hypoxia. Unlike prior work, however, we found that cardiac function improvement after MI due to normoxic low altitude simulation treatment was not associated with a HIF1a mediated responses (Fig 3) or neovascularization [18]. Our findings are novel in that previous studies have only evaluated the therapeutic benefit of altitude simulation to elevations high enough to cause hypoxia [18].

Left ventricular function was improved by normoxic low altitude simulation corresponding to a low reduction of 40 mmHg in barometric pressure, from 754 mmHg to 714 mmHg for 3 hours a day over a week (Fig 1B). This was evident through M- mode echocardiograms showing a lack of anterior LV wall movement in animals that did not receive normoxic low altitude simulation (Fig 1A). Infarct size was also 11% smaller in treated mice than in untreated mice (Fig 2F). These results are similar in magnitude to the benefit seen in other trials of pharmacologic and non-pharmacologic therapies for MI in humans. For example, the METOCARD-CNIC trial reported a roughly 20% reduction in infarct size in human MI patients with ST-segment-elevations undergoing primary percutaneous coronary intervention who had received 15 mg of β_1_ adrenergic blocker metoprolol before reperfusion [19, 20]. The effect size for normoxic low altitude simulation is similar to that achieved by other experimental therapies.

The benefit we observed from normoxic low altitude simulation post-MI does not appear to be dependent on the HIF 1a activation pathway (Fig 3). HIF-1α is often viewed as a “helpful” agent, being activated in hypoxic conditions to allow the ischemic tissue to better deal with lowered oxygenation. HIF-1α has been implicated in the induction of angiogenesis, limiting infarct size, and improving myocardial function after acute coronary occlusion in mice [21]. We found reduced expression of HIF-1α (Fig 3C) after MI in the peri-infarct tissue of animals treated with normoxic low altitude simulation after LAD ligation surgery compared to the controlgroup animals. As HIF1a up-regulation is increased linearly with the level of hypoxia [22], a reduction in penumbra HIF-1α expression after normoxic low altitude simulation treatment points to less hypoxia in the ischemic zone. We found that no significant differences exist in the microvascular networks between the control and intervention group when assessed by lectin perfusion *ex vivo* (Fig 4). This implies that the improvement in cardiac function that we have observed in normoxic low altitude simulation treated mice after 7 days of treatment is not due to neovascularization. Taken together, our data suggest that that normoxic low altitude simulation treatment may lead to improvements in perfusion, and therefore oxygenation, without neovascularization.

We found that in mice exposed to normoxic low altitude simulation, stroke volume and cardiac output were increased while total systemic vascular resistance was reduced by over 20% with acute barometric pressure reductions of 40 mmHg (from 754 mmHg to 714 mmHg). This drop in systemic vascular resistance, and subsequent increase in cardiac output and stroke volume, could be due to an overall increase in distention of the resistance arteries under lowered barometric pressures. There are pharmacologic agents that have set this precedent. For example, administration of Urocortin II, an endogenous peptide with vasodilatory and inotropic cardiac effects, shows a significant increase in cardiac output and stroke volume while decreasing arterial resistance without a change in heart rate of healthy mice [23].

We found that normoxic low altitude simulation can act alone to increase vascular diameter of arterial segments ex vivo (Fig 8) with or without the aid of major endogenous endothelial factors. Inhibition of nitric oxide and prostaglandin activity does not significantly dampen vessel diameter increases induced by a reduction in barometric pressure (Fig 9). This finding in *ex vivo* arterial segments mounted on a pressure myograph system corroborated the reduced systemic vascular resistance *in vivo* in our mouse model.

### Limitations and strengths

A limitation of our study is the lack of measurement of the partial pressure of oxygen inside our chamber when low altitude was simulated in our custom constructed hypobaric chamber. Overall, minor reductions in levels of oxygen saturation corresponding to the barometric pressures that we used for normoxic low altitude simulation (corresponding to either 714 mmHg and 674 mmHg) generally do not constitute a hypoxic environment for mammalian tissues [24–26]. Moreover, in the case of the catheterized *in vivo* mice, these animals were breathing 100% oxygen during the experiment, excluding hypoxia as a contributing factor during these experiments. In addition, as our experiments evaluated acute responses, and we cannot exclude chronic adaptations that would attenuate or reverse these changes. Another limitation to our study is that we do not measure any effects of chronic exposure to normoxic low altitude simulation or acclimatization to normoxic low altitude simulation. Our current study aimed to explore normoxic low altitude simulation as a therapeutic tool, so we evaluated acute responses and cannot be sure of any chronic effects of normoxic low altitude simulation that may diminish or reverse the changes we observed. It is possible that the effects of longer-term exposures to normoxic low altitude simulation could hinder the therapeutic benefit of the air pressure reduction.

Strengths of this work include pharmacological dissection of the observed changes in lumen diameter to exclude most endothelium-dependent components of this phenomenon and obtaining similar observations of these changes using invasive hemodynamic measurements in an intact *in vivo* model. Additionally, the use of a chamber control group in the LAD ligation model shows that any benefit observed in the treatment group was not due to factors like differential handling between the groups of mice. Another strength of the work is clear cut demonstration of the therapeutic benefit using a clinically relevant model of MI, the LAD ligation model, as measured by clinically relevant techniques, including echocardiography. Moreover, since altitude chambers are commercially available, our findings could be translated into humans in a straightforward way.

### Clinical implications

Our data show that normoxic low altitude simulation can improve ventricular function and reduce infarct size after MI. This may explain the epidemiological studies showing that individuals living at higher altitudes have lower risk of MI and stroke. We found that normoxic low altitude simulation can reduce systemic vascular resistance and increase cardiac output and may increase perfusion to ischemic tissue without concomitant neovascularization. Since the therapeutic approach to acute ischemic disorders like MI and ischemic stroke involve improving blood flow to ischemic tissues, or reperfusion [27], a non-invasive mechanical technique of normoxic low altitude simulation might be useful as a therapeutic tool to improve blood flow to ischemic tissues. Similarly, since the therapeutic approach for managing congestive heart failure is afterload reduction [28], normoxic low altitude simulation could have therapeutic benefits, at least acutely. Altitude simulation is a current and commercially viable everyday reality, occurring in passenger aircraft [29, 30], and negative pressure hospital rooms [31]. There is compelling evidence that commercial airline personnel have lower mortality from MI [32, 33]which further paves the way for interesting discussion of any therapeutic benefit to reductions in atmospheric pressure. Therefore, further preclinical work evaluating hypobaric exposure, with or without supplemental oxygen, may be justified. This work may have potential for clinical translations to humans with MI.

## Supporting information

S1 Fig Experimental animals were placed within a specially constructed chamber to simulate low altitude. The animals were exposed to 40 mmHg air pressure reductions for a period of 3 hours daily for 1 week post the LAD ligation surgery to study the therapeutic potential of normoxic low altitude simulation on cardiac function after MI.
(TIF)

S2 Fig Pressure volume loops obtained in healthy mice in reduced pressure conditions. Pressure-volume loops were obtained using a conductance catheter placed in the left ventricle of anaesthetized intact mice breathing oxygen-enriched air inside a hypobaric chamber.
(TIF)

S3 Fig Arterial responses to decreases in pressure using a pressure myograph. Segments of murine mesenteric artery were placed in a pressure myograph which was in turn placed within a specially constructed chamber to allow reductions in barometric pressure (i.e. low altitude simulation–normoxic low altitude simulation).
(TIF)

S4 Fig Representative echocardiogram baseline images are shown. The images shown were obtained one day before after the LAD ligation.
(TIF)

S5 Fig Hemodynamic parameters were obtained through echocardiography for experimental and untreated animals (both chamber and shelf controls) after LAD ligation surgery. There were no significant differences in heart rate (a) in all sets of animals from Day 1 to Day 7. There were statistically significant increases in fractional shortening (b), ejection fraction (c) stroke volume (d) and cardiac output (e) in animals that received 3 hours of normoxic low altitude simulation (at 714 mmHg) for 7 days in comparison to shelf and chamber control animals. Improvements in left ventricular function as shown by these parameters were absent from animals that did not receive normoxic low altitude simulation treatment. There are no significant differences between the chamber and shelf control groups at any parameters.
(TIF)

S1 Dataset This dataset includes all the preliminary data presented in the results section of this manuscript. (XLSX)

## Abbreviations

PSS: Physiological salt solution
PE: Phenylephrine
Mch: Methacholine
NO: Nitric Oxide
SV: Stroke volume
CO: Cardiac output
MI: Myocardial infarction
