# Supplementary figures and images for: Low altitude simulation without hypoxia improves left ventricular function after myocardial infarction by reducing ventricular afterload
Source: PLoS One. 2019 May 31;14(5):e0215814. doi: 10.1371/journal.pone.0215814 (PMC6544215; doi:10.1371/journal.pone.0215814)

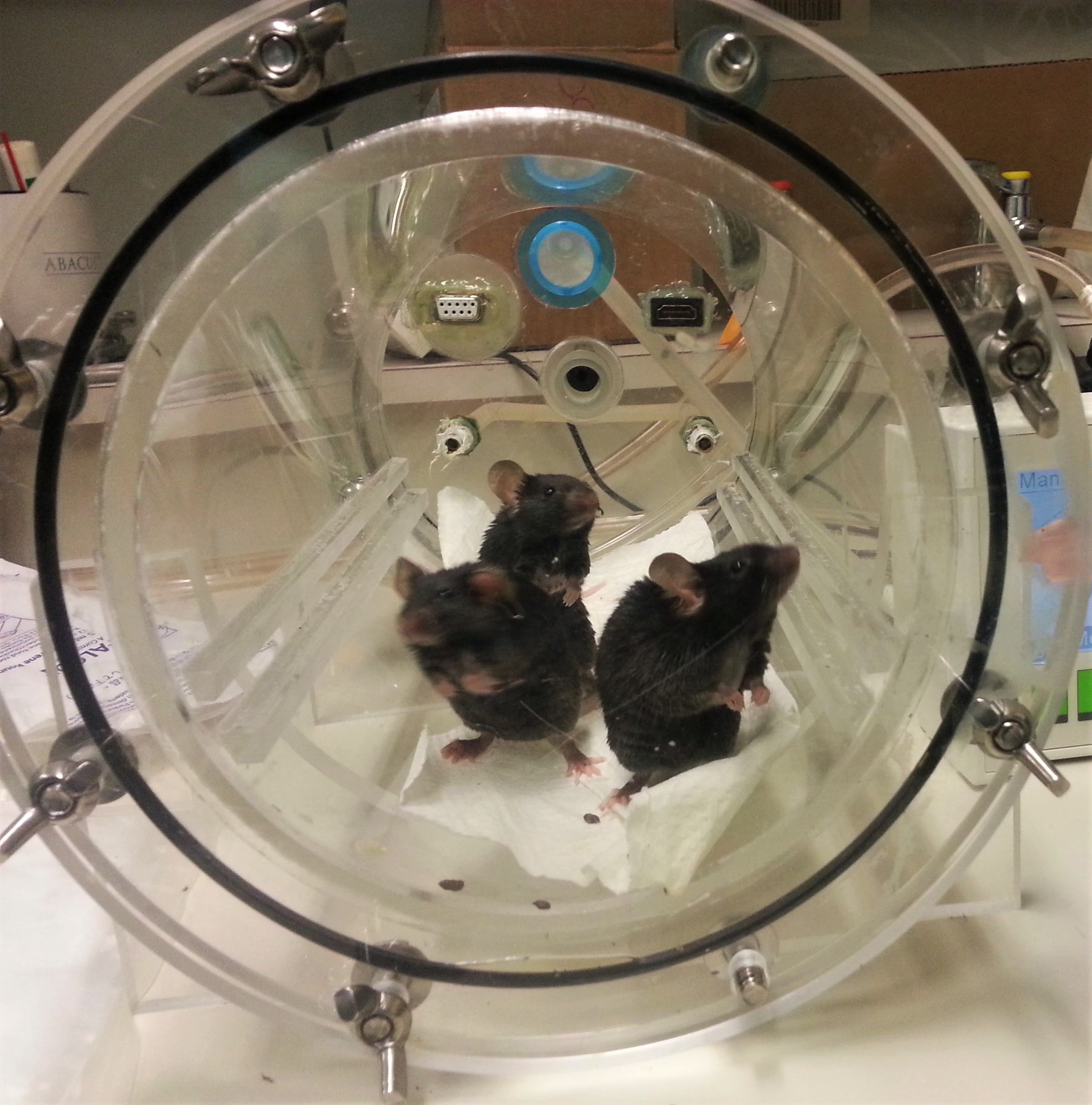

Supplement: S1 Fig — The animals were exposed to 40 mmHg air pressure reductions for a period of 3 hours daily for 1 week post the LAD ligation surgery to study the therapeutic potential of normoxic low altitude simulation on cardiac function after MI. (TIF) [file pone.0215814.s001.TIF]

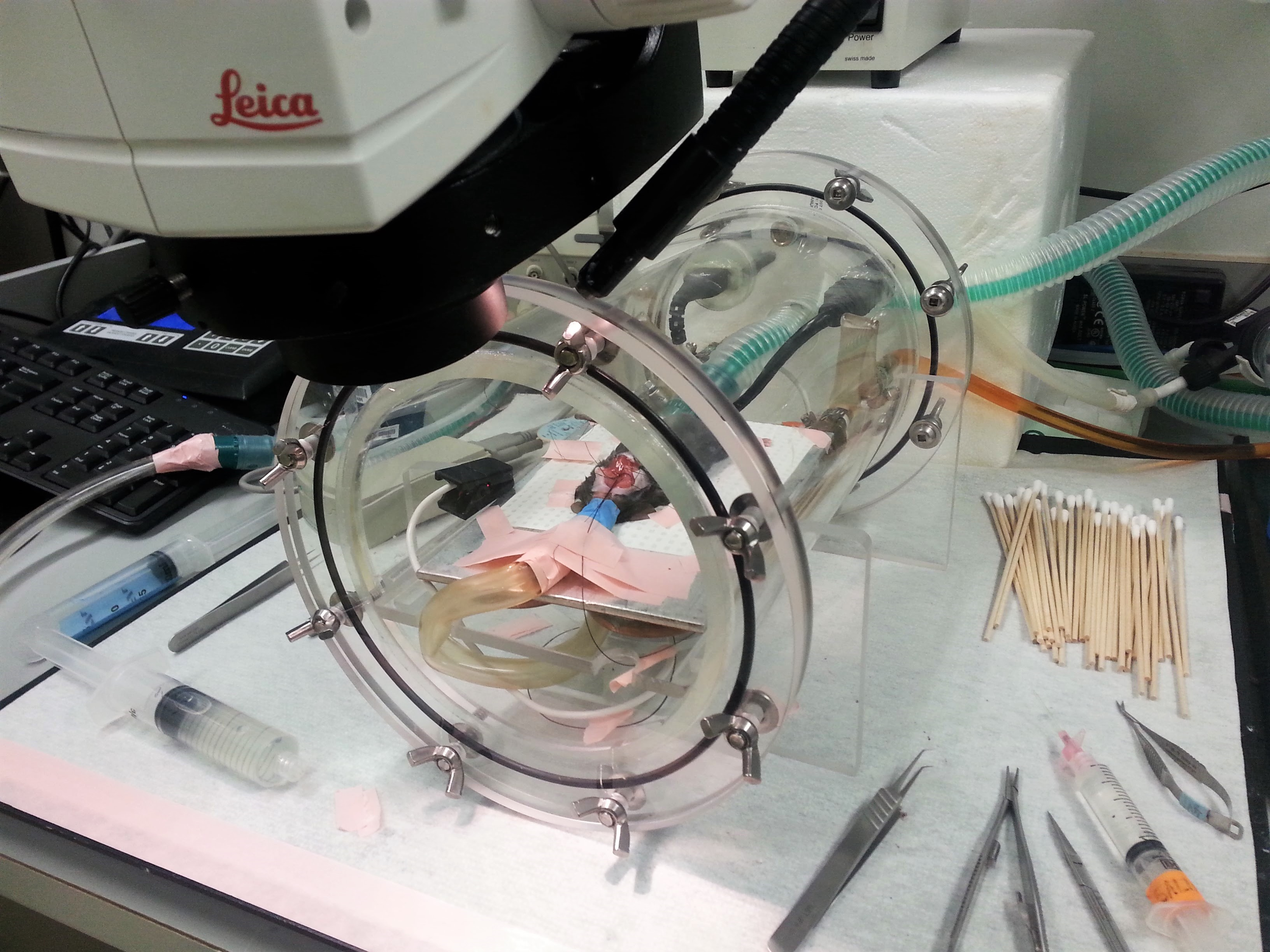

Supplement: S2 Fig — Pressure-volume loops were obtained using a conductance catheter placed in the left ventricle of anaesthetized intact mice breathing oxygen-enriched air inside a hypobaric chamber. (TIF) [file pone.0215814.s002.TIF]

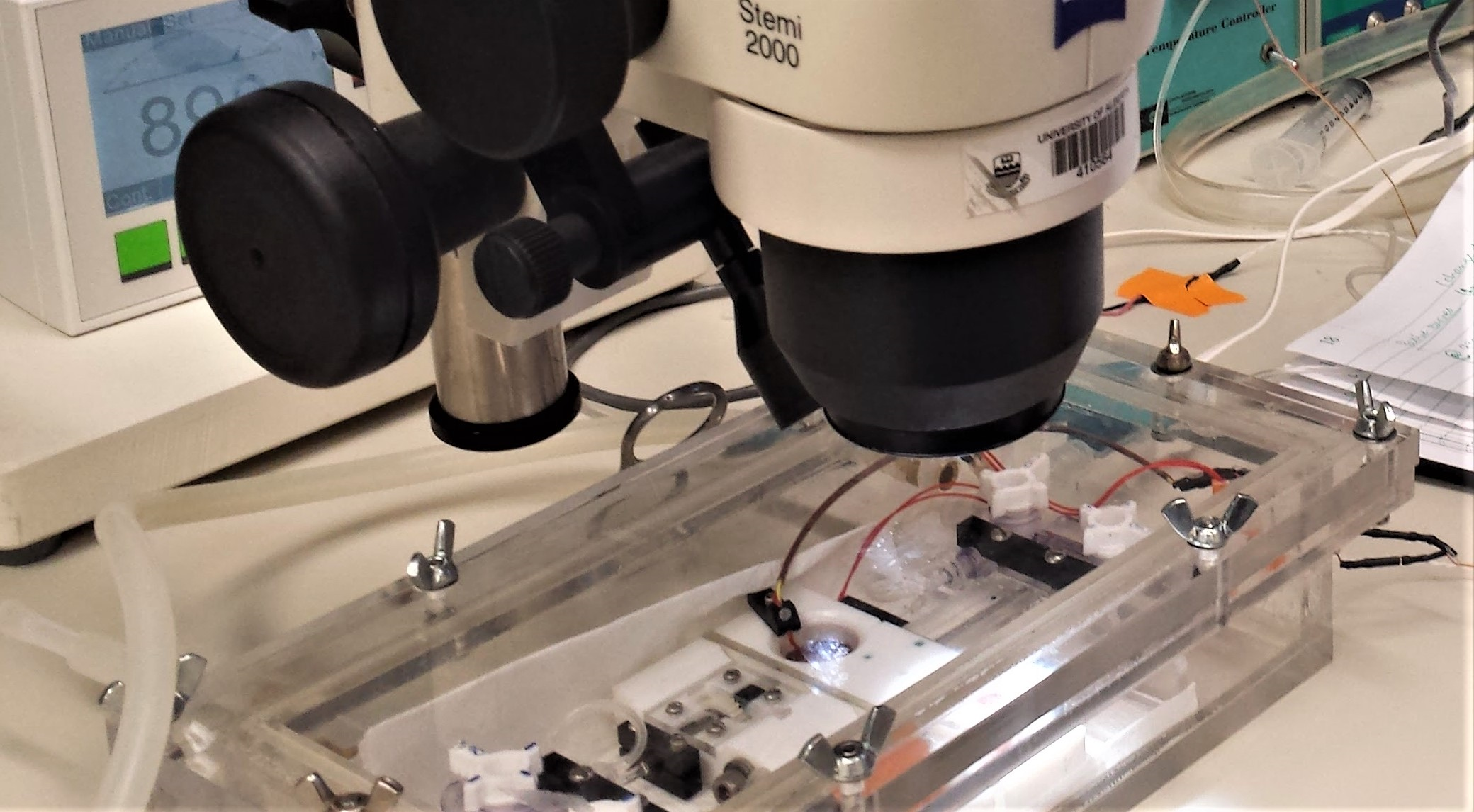

Supplement: S3 Fig — Segments of murine mesenteric artery were placed in a pressure myograph which was in turn placed within a specially constructed chamber to allow reductions in barometric pressure (i.e. low altitude simulation–normoxic low altitude simulation). (TIF) [file pone.0215814.s003.TIF]

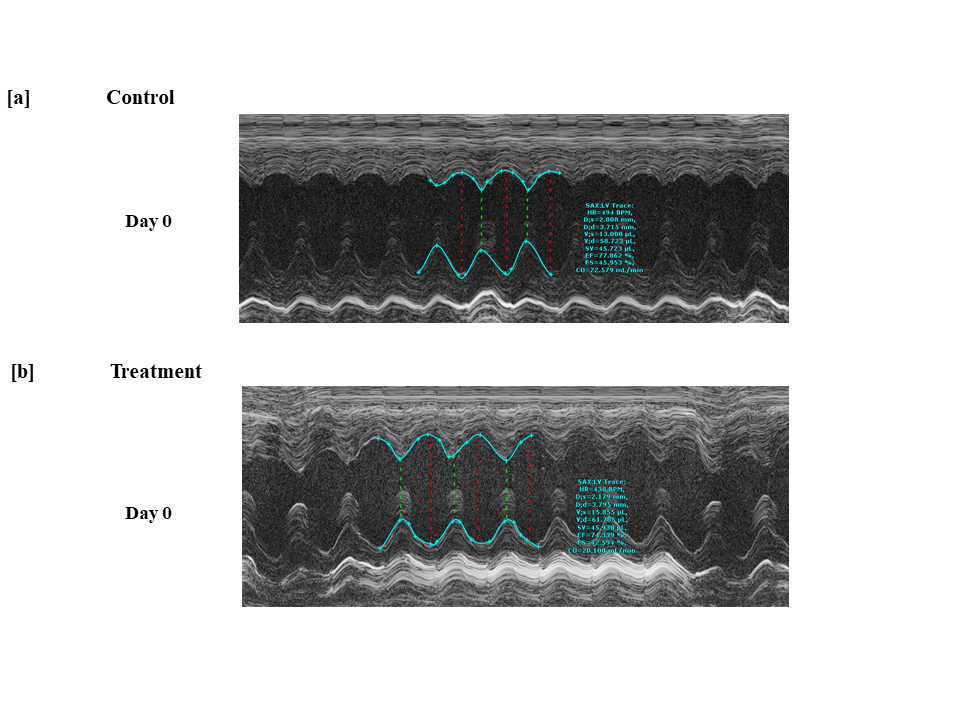

Supplement: S4 Fig — The images shown were obtained one day before after the LAD ligation. (TIF) [file pone.0215814.s004.TIF]

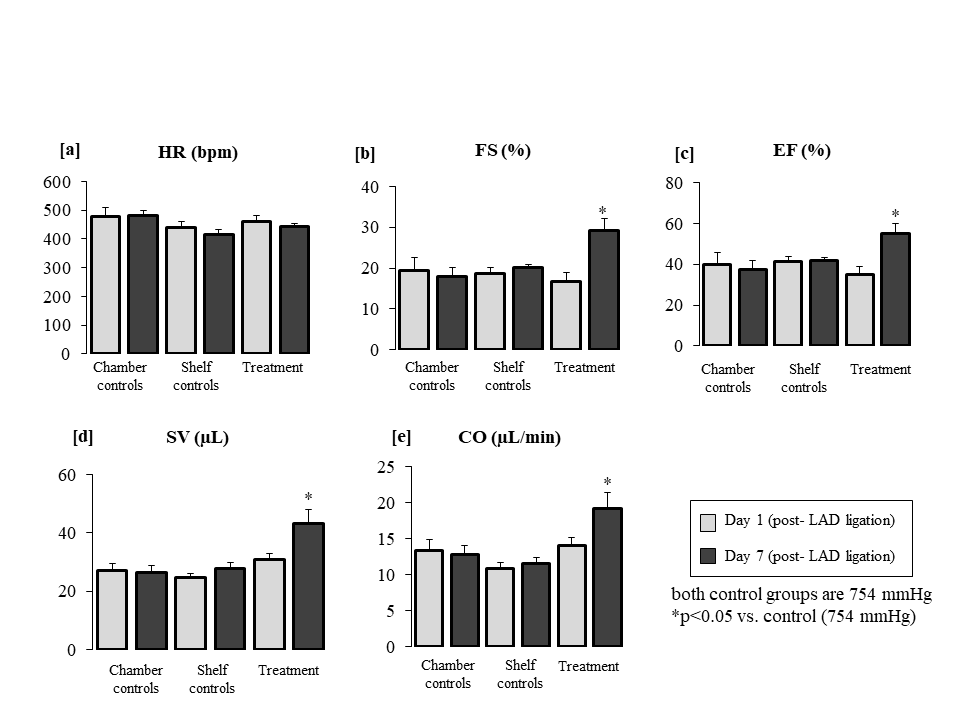

Supplement: S5 Fig — There were no significant differences in heart rate (a) in all sets of animals from Day 1 to Day 7. There were statistically significant increases in fractional shortening (b), ejection fraction (c) stroke volume (d) and cardiac output (e) in animals that received 3 hours of normoxic low altitude simulation (at 714 mmHg) for 7 days in comparison to shelf and chamber control animals. Improvements in left ventricular function as shown by these parameters were absent from animals that did not receive normoxic low altitude simulation treatment. There are no significant differences between the chamber and shelf control groups at any parameters. (TIF) [file pone.0215814.s005.TIF]
